# Supplementary material for: The interactome of intact mitochondria by cross-linking mass spectrometry provides evidence for coexisting respiratory supercomplexes
Source: Mol Cell Proteomics. 2017 Dec 8;17(2):216–32. doi: 10.1074/mcp.RA117.000470 (PMC5795388; doi:10.1074/mcp.RA117.000470)
Supplement: Supplemental Data [file supp_RA117.000470_133922_0_supp_23978_fzffwf.zip › spectra_annotation/mito_spectra_annotation/359-1-6-1-12-1.pdf]

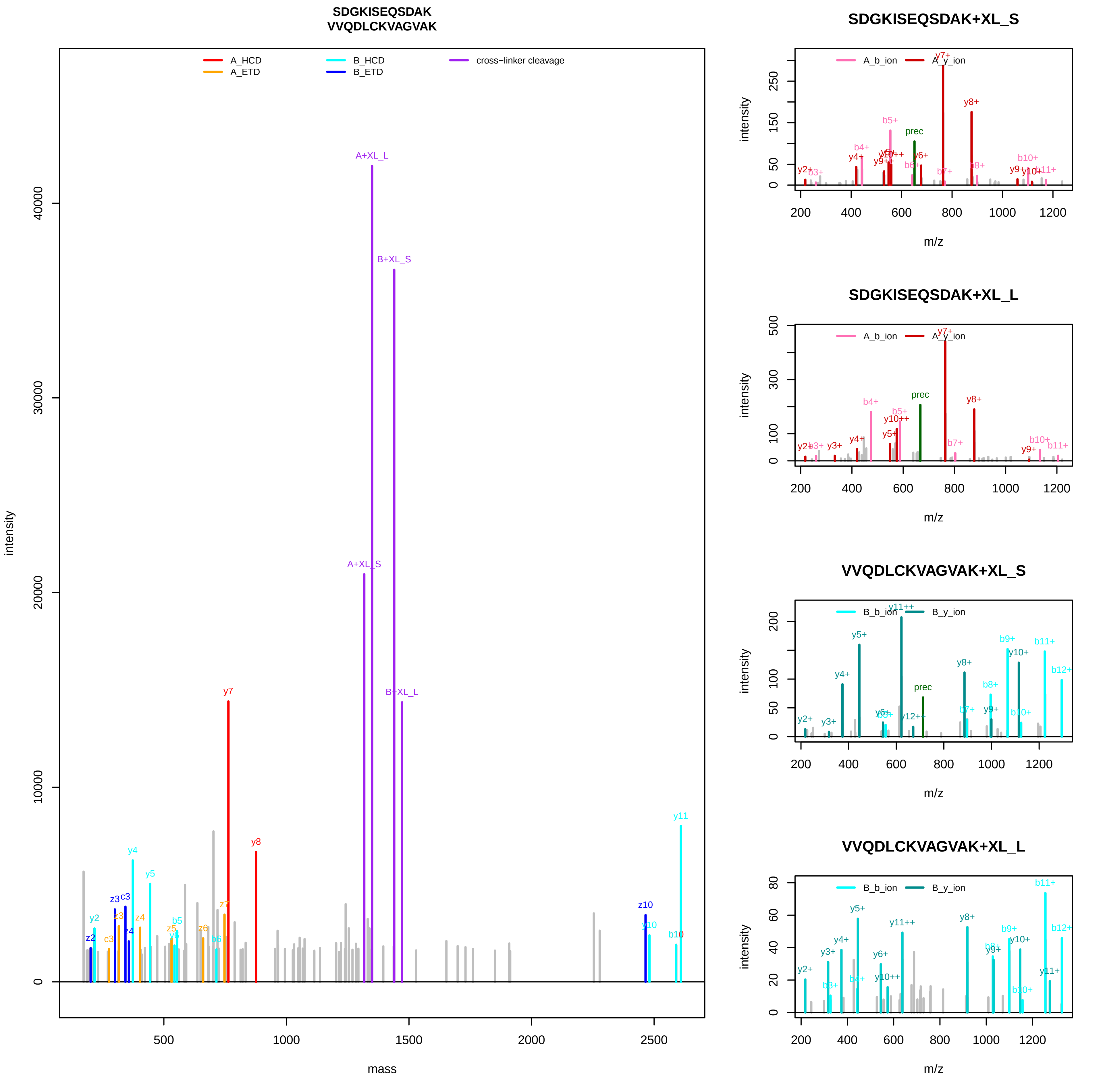

SDGKISEQSDAK
VVQDLCKVAGVAK
A_HCD
A_ETD
B_HCD
B_ETD
cross−linker cleavage
A+XL_L
B+XL_S
A+XL_S
B+XL_L
y7
y8
y11
y10
z10
b10
y2
y4
y5
b5
z2
z3
c3
z4
z4
z5
z6
z7
c3
b6
intensity
mass
0
10000
20000
30000
40000
500
1000
1500
2000
2500
SDGKISEQSDAK+XL_S
A_b_ion
A_y_ion
y7+
y8+
b5+
prec
b4+
y4+
y2+
b3+
y6+
b6+
b7+
b8+
b10+
y9+
y10+
b11+
SDGKISEQSDAK+XL_L
y3+
y5+
y10++
VVQDLCKVAGVAK+XL_S
B_b_ion
B_y_ion
y11++
y12++
b8+
b9+
b11+
b12+
VVQDLCKVAGVAK+XL_L
m/z
200
400
600
800
1000
1200
